# Sirt3 is essential for apelin-induced angiogenesis in post-myocardial infarction of diabetes

**DOI:** 10.1111/jcmm.12453

**Published:** 2014-10-14

**Authors:** Xuwei Hou, Heng Zeng, Xiaochen He, Jian-Xiong Chen

**Affiliations:** Department of Pharmacology and Toxicology, University of Mississippi Medical CenterJackson, MS, USA

**Keywords:** sirtuin 3, angiogenesis, apelin, apoptosis, myocardial infarction, diabetes

## Abstract

Heart failure following myocardial infarction (MI) is the leading cause of death in diabetic patients. Angiogenesis contributes to cardiac repair and functional recovery in post-MI. Our previous study shows that apelin (APLN) increases Sirtuin 3 (Sirt3) expression and ameliorates diabetic cardiomyopathy. In this study, we further investigated the direct role of Sirt3 in APLN-induced angiogenesis in post-MI model of diabetes. Wild-type (WT) and Sirt3 knockout (Sirt3KO) mice were induced into diabetes by i.p. streptozotocin (STZ). STZ mice were then subjected to MI followed by immediate intramyocardial injection with adenovirus-apelin (Ad-APLN). Our studies showed that Sirt3 expression was significantly reduced in the hearts of STZ mice. Ad-APLN treatment resulted in up-regulation of Sirt3, angiopoietins/Tie-2 and VEGF/VEGFR2 expression together with increased myocardial vascular densities in WT-STZ+MI mice, but these alterations were not observed in Sirt3KO-STZ+MI mice. *In vitro*, overexpression of APLN increased Sirt3 expression and angiogenesis in endothelial progenitor cells (EPC) from WT mice, but not in EPC from Sirt3KO mice. APLN gene therapy increases angiogenesis and improves cardiac functional recovery in diabetic hearts *via* up-regulation of Sirt3 pathway.

## Introduction

Myocardial angiogenesis is a process of forming new vessels to provide oxygen and nutrient supply to the ischaemic area of myocardial infarction (MI), which is a key adaptive mechanism to restore blood perfusion and a key determinant of infarct size expansion in post-MI [1,2]. Improvement of angiogenesis is being considered as an innovative therapeutic approach for the treatment of ischaemic heart disease [3,4]. Diabetes mellitus (DM) is characterized by hyperglycaemia, which leads to extensive cardiovascular complications including impairment of angiogenesis [5,6]. Coronary artery disease (CAD) is one of the major complications of DM [6]. Epidemiological studies revealed that myocardial ischaemia/infarction is the leading cause of morbidity and mortality in the patients with DM [7,8]. A population-based study also showed that the incidence of MI in diabetic patients is significantly higher than non-diabetic patients [9]. Previously, we have shown that DM impairs myocardial angiogenesis *via* disruption of angiopoietins/Tie-2 and suppression of VEGF expression [10,11]. DM-associated impairment of angiogenesis contributes to the exacerbation of ischaemic injury and heart failure of diabetes [10,12,13]. Therefore, it is urgent to develop new agents for the treatment of impaired myocardial angiogenesis and post-MI heart failure in DM.

Apelin (APLN) is a bio-activated peptide with a potent angiogenic activity [14]. APLN exerts its biological effect *via* binding to the APLN receptor (APJ). APLN has been indicated as a key regulator of angiogenesis in different tissues [15,16]. A recent study shows that deficiency of APLN exacerbates MI adverse remodelling and ischaemia-reperfusion injury [17]. Our previous study shows that treatment with APLN promotes myocardial angiogenesis and improves cardiac function in post-MI mice [18]. Our recent study further demonstrated that treatment with bone marrow cells overexpressing APLN enhances myocardial angiogenesis and functional recovery, accompanied by increased Sirt3 levels in the ischaemic heart [19]. These findings indicate that Sirt3 may have a critical role for APLN-mediated cardiac protection in post-MI. Sirt3 is a member of a highly conserved family of protein deacetylases, which is closely associated with the prolonged lifespan of human [20]. Sirt3 has been attracted much attention because it regulates cardiomyocyte apoptosis, survival and cardiac hypertrophy [21–23]. So far, the direct link between APLN and Sirt3 in the regulation of myocardial angiogenesis in post-MI diabetes has not been reported.

This study was designed to evaluate the direct functional role of Sirt3 in APLN-mediated angiogenesis in diabetic mouse model. Wild-type (WT) and Sirt3 knockout (Sirt3 KO) mice were treated with streptozotocin (STZ) to induce hyperglycaemic DM model followed by MI by ligation of left anterior descendant artery (LAD). Using this ischaemic STZ mouse model, we have examined the effects of APLN gene therapy on the ischaemia-induced angiogenesis in diabetic mice. Moreover, we have explored the potential mechanisms by which Sirt3 regulates APLN-induced myocardial angiogenesis in diabetes.

## Materials and methods

All procedures conformed with the Institute for Laboratory Animal Research Guide for the Care and Use of Laboratory Animals and were approved by the Animal Care and Use Committee of University of Mississippi Medical Center (protocol identifier: 1280). The investigation conformed to the National Institutes of Health (NIH, Bethesda, Maryland ) Guide for the Care and Use of Laboratory Animals (NIH Pub. No. 85-23, Revised 1996).

### Experimental animal model and treatment

Wild-type control and Sirt3 knockout (Sirt3KO) mice (obtained from Jackson laboratory, Bar Harbor, ME, USA) were bred by our laboratory. Experimental mice (male at 4–5-month age) were intraperitoneally (i.p) injected with STZ (50 mg/kg, Sigma-Aldrich Co, MO, USA) for 5 days to induce diabetes [13]. At 6 weeks, mice with blood glucose level >300 mg/dl were selected for the left anterior descending coronary artery (LAD) ligation to induce MI [10,12,13,18,19]. Ischaemic areas were intramyocardial injected with adenovirus-APLN (Ad-APLN) and adenovirus-β-gal adenovirus (Ad-β-gal) at the dose of 1 × 10^9^ PFU per heart at four sites. Experimental STZ mice were divided into four groups: (*i*) WT-STZ + Ad-β-gal; (*ii*) WT-STZ + Ad-APLN; (*iii*) SIRT3KO-STZ + Ad-β-gal; and (*iv*) SIRT 3KO-STZ + Ad-APLN. After 2 weeks of ad-APLN or Ad-β-gal gene therapy, mice were killed by cervical dislocation under anaesthesia with isoflurane.

### Echocardiography

Transthoracic echocardiograms were performed on STZ mice at 2 weeks after LAD ligation using a Vevo770 Imaging System (VisualSonics Inc, Canada). Left ventricle ejection fraction (EF) and fractional shortening (FS) were recorded along with LV cavity dimensions (end-diastolic and end-systolic).

### Myocardial capillary and arteriole densities

Heart sections (10 μm) were incubated with primary antibodies. Capillary was labelled with fluorescein-labelled Griffonia Bandeiraea simplicifolia isolectin B4 (IB4, 1:50; Molecular Probe, Invitrogen, USA) or vWF and arteriole was stained by anti-α-smooth muscle actin (α-SMA; 1:100; Sigma-Aldrich). The number of capillary and arteriole was counted using image-analysis software (Image J, NIH) and expressed as capillary density per field [10,12,13,18,19].

### Endothelial progenitor cells treatment and transfection

Endothelial progenitor cells (EPC) was isolated and cultured from bone marrow of WT and Sirt3KO mice as described previously [12,13,24]. Two EPC markers, IB4 and CD34, were used for EPC identification by immunohistochemistry. To mimic *in vivo* hyperglycaemic conditions of DM model, EPC were exposed to high glucose (30 mmol/l) for 24 hrs, and followed by transfection with Ad-APLN and Ad-β-gal (1 × 10^9^ PFU) in serum-free medium.

### EPC tube formation

Endothelial progenitor cells (4.5 × 10^4^ cells/well) were seeded on the layer of polymerized Matrigel (BD Biosciences, Bedford, MA, USA), followed by incubation for 6 hrs. Tube formation was quantified by measuring the long axis of each tube in five random fields per well using image-analysis software (Image J, NIH).

### EPC proliferation assay

Endothelial progenitor cells (5 × 10^3^ cells/well) were incubated in 96-well plates to allow growth for 96 hrs. Cells were then incubated with 3-(4,5-Dimethylthiazol-2-yl)-2,5-diphenyltetrazoliumbromide (MTT, 0.5 mg/ml) for 4 hrs. Formazan crystal was lysed by dimethyl sulfoxide, and absorbance was measured at 562 nm with ELISA reader (BioTek).

### Western blot analysis

Hearts were harvested and homogenized in lysis buffer for Western analysis. The membranes were blotted with APLN, VEGFR2, Tie-2 (1:1000; Cell Signaling, MA, USA), VEGF, Ang-2 and Ang-1 (1:1000; Sigma-Aldrich) antibodies. Akt and eNOS phosphorylation were measured by phosphorylated Akt and eNOS antibodies followed by total Akt and eNOS antibodies. The membranes were then washed and incubated with a secondary antibody coupled to horseradish peroxidase and densitometric analysis was carried out using image acquisition and analysis software (TINA 2.0).

### Terminal deoxynucleotidyl transferase dUTP nick end labelling (TUNEL) assay

The apoptotic cells in heart tissue and cultured cells were detected by *in situ* DeadEndTM Colorimetric Apoptosis Detection System (Promega, Madison, WI, USA) according to the manufacturer's instructions. The sections were counterstained with DAPI. Apoptosis was indexed by counting TUNEL^+^ cells per 100 nuclei per section. vWF was co-stained with TUNEL.

### Statistical analysis

Data are presented as the mean ± SD. Statistical analysis of data was performed with one-way anova followed by the *post hoc* test and *P* < 0.05 were considered as significant.

## Results

### APLN gene therapy on Sirt3 expression in WT-STZ+MI and Sirt3KO-STZ+MI mice

**Fig. 1:**
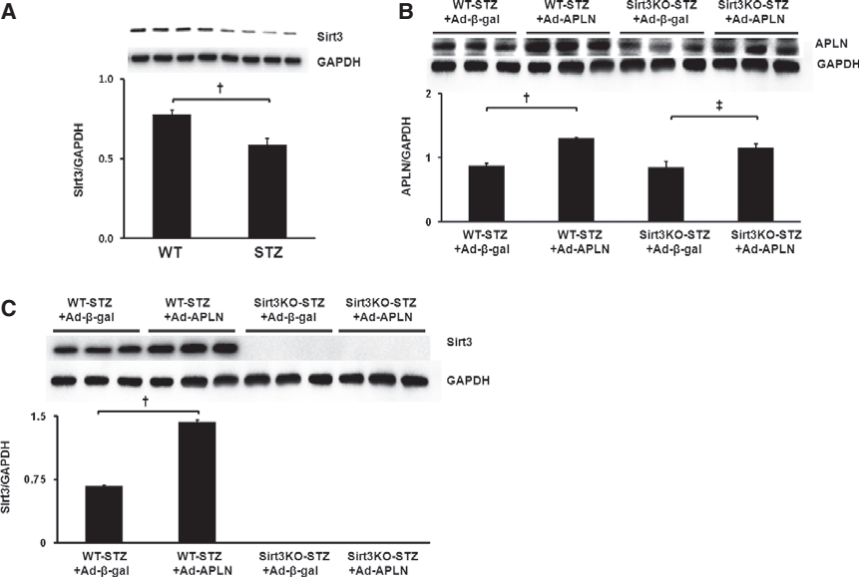
APLN gene therapy on Sirt3 expression in post-MI WT and Sirt3KO-STZ mice. (**A**) Sirt3 expression was significantly reduced in the STZ mouse heart compared to non-diabetic mouse heart (*n* = 4 mice). (**B**) Ad-APLN treatment significantly increased APLN expression in the heart of WT-STZ and Sirt3KO-STZ mice (*n* = 3, †*P* < 0.001, ‡<0.05). (**C**) Ad-APLN gene therapy increased Sirt3 expression in the heart (*n* = 3, †*P* < 0.001). Ad-APLN gene therapy did not induce Sirt3 expression in the hearts of Sirt3KO-STZ mice.

We first examined Sirt3 levels in the hearts of SIZ mice. Our Western blot analysis showed that Sirt3 expression was significantly reduced in STZ mice (Fig. 1A). Intramyocardial injection with Ad-APLN resulted in a significant increase in APLN expression in the hearts of WT-STZ and Sirt3KO-STZ mice (Fig. 1B). This was accompanied by a significant increase in Sirt3 expression in the hearts of WT-STZ mice, but not in Sirt3KO-STZ mice (Fig. 1C).

### APLN gene therapy increases expression of angiogenic growth factors and neovascularization in STZ+MI mice

**Fig. 2:**
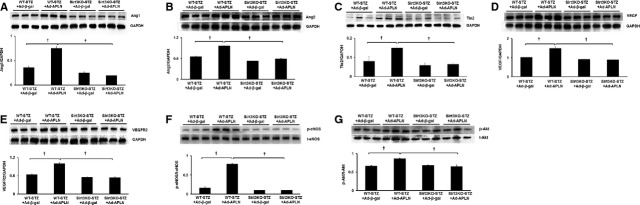
APLN gene therapy on angiogenic growth factors and angiogenesis in post-MI STZ mice. (**A**–**C**) Ad-APLN gene therapy increased expression of Ang-1 (**A**), Ang-2 (**B**) and Tie-2 (**C**) in the heart of WT-STZ mice; these changes were abolished in Sirt3KO-STZ mice. In Ad-APLN treatment groups, WT-STZ mice had higher Ang-1, Ang-2 and Tie-2 expression levels than Sirt3KO-STZ mice (*n* = 3, †*P* < 0.001). (**D** and **E**) The expression of VEGF (**D**) and VEGFR2 (**E**) was significantly increased in WT-STZ mouse hearts, but these increases were not observed in hearts of Sirt3 KO-STZ mice (*n* = 3, †*P* < 0.001). In mice treated with Ad-APLN, WT-STZ mice had higher VEGF and VEGFR2 expressions than Sirt3KO-STZ mice (*n* = 3, †*P* < 0.001). (**F** and **G**) p-eNOS was significantly increased in WT-STZ mice treated with Ad-APLN compared to mice treated with Ad-β-gal (*n* = 3, †*P* < 0.001). No changes were observed in Sirt3 KO-STZ mice treated with Ad-APLN. Increased p-Akt levels were found in the heart of WT-STZ mice (*n* = 3, †*P* < 0.05), but not Sirt3KO-STZ mice. WT-STZ treated with Ad-APLN had higher p-eNOS and p-Akt levels than Sirt3KO-STZ mice with Ad-APLN treatment (*n* = 3, †*P* < 0.001).

**Fig. 3:**
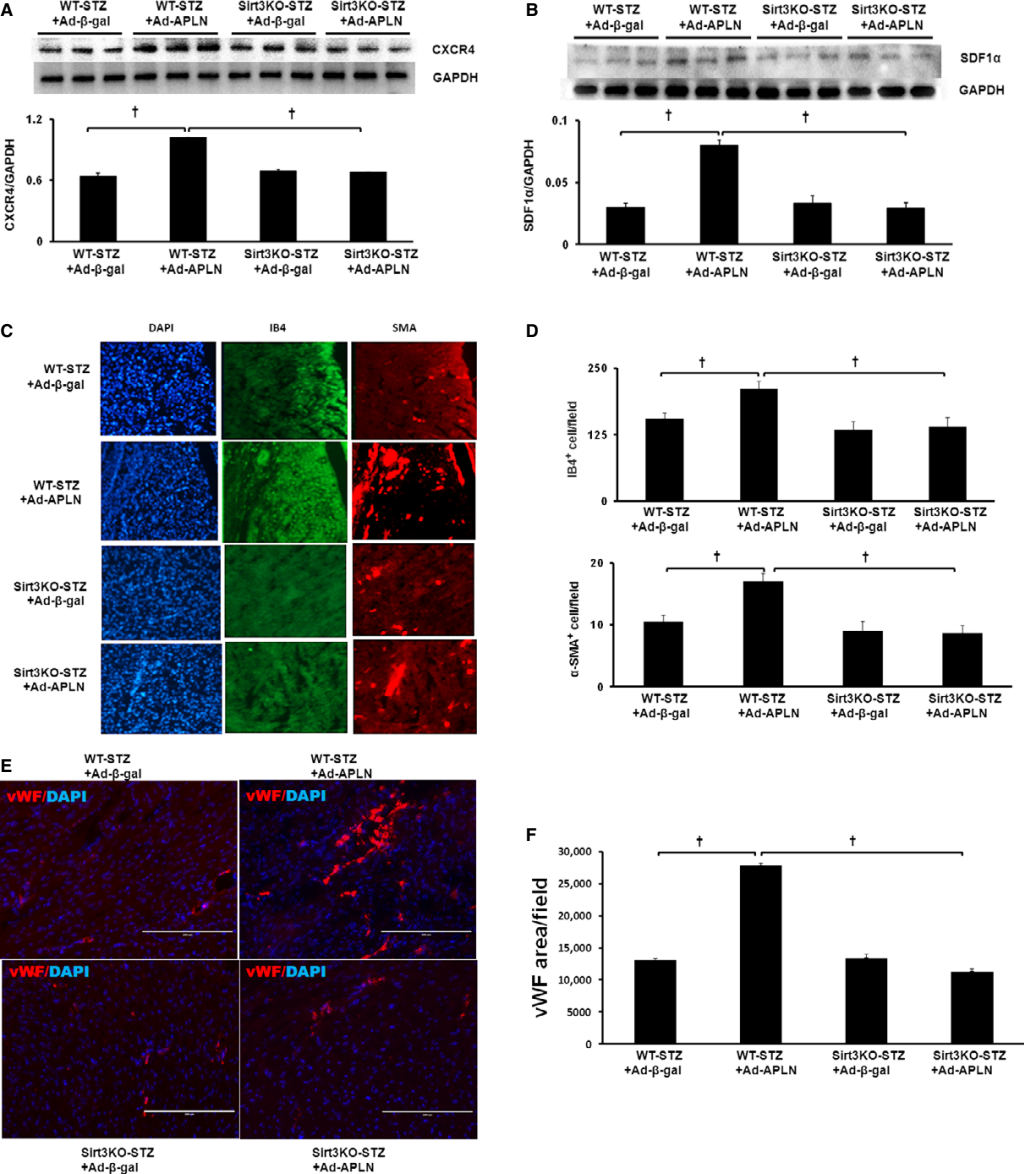
APLN gene therapy on expression of EPC chemotactic factor and *in situ* angiogenesis in the heart of STZ mice. (**A** and **B**) Ad-APLN gene therapy resulted in significant up-regulation of CXCR4 (**A**) and SDF1 (**B**) expression in post-MI WT-STZ mice, whereas APLN did not change CXCR4 or SDF1 expression in Sirt3KO-STZ mice compared to Ad-β-gal. Within the Ad-APLN-treated mice, CXCR4 and SDF1 expression was significantly higher in WT-STZ mice than that of Sirt3KO-STZ mice (*n* = 3 mice, †*P* < 0.001). (**C** and **D**) The capillaries were labelled by IB4 staining (green) and arterioles were labelled by α-SMA (red). Ad-APLN-treated WT-STZ mice displayed higher IB4-positive and α-SMA-positive cells in the heart compared to Sirt3KO-STZ mice. In WT-STZ mice, Ad-APLN gene therapy increased capillary and arteriole densities in the border zone of ischaemia compared to Ad-β-gal treatment. Knockout of Sirt3 dramatically attenuated Ad-APLN-induced myocardial angiogenesis in STZ mice (*n* = 5 mice/group, †*P* < 0.001). (**E** and **F**) Capillary was labelled with vWF (green). vWF area was measured in the border zone of ischaemia. Ad-APLN treatment significantly increased capillary density in WT-STZ, mice but failed to increase capillary density in Sirt3KO-STZ mice (*n* = 5 mice/group, †*P* < 0.001)

In WT-STZ+MI mice, APLN gene therapy significantly increased expression of Ang-1, Ang-2 and Tie-2 in the ischaemic hearts (Fig. 2A–C). Overexpression of APLN led to significant increases in VEGF and VEGFR2 expression in WT-STZ+MI mouse hearts (Fig. 2D and E). Moreover, Akt and eNOS phosphorylation was significantly increased in WT-STZ+MI mice treated with Ad-APLN compared to WT-STZ+MI received Ad-β-gal (Fig. 2F and G). In Sirt3KO-STZ+MI mice, Ad-APLN treatment has little effect on the expression of angiopoietins/Tie-2 and VEGF/VEGFR2 as well as levels of Akt/eNOS phosphorylation (Fig. 2A–G). CXCR-4 and SDF-1α expression was also significantly up-regulated by Ad-APLN treatment in post-MI STZ mice (Fig. 3A and B). Knockout of Sirt3 in STZ+MI mice further blunted APLN-induced CXCR-4 and SDF-1α expression (Fig. 3A and B). Ad-APLN treatment significantly increased capillary (IB4 and vWF) density and arteriole density in the border zone of ischaemia as compared to WT-STZ+MI mice treated with Ad-β-gal (Fig. 3C–F). However, APLN gene therapy did not increase capillary and arteriole densities in Sirt3KO-STZ+MI mice (Fig. 3C–F).

### APLN gene therapy attenuates myocardial apoptosis in post-MI STZ mice

**Fig. 4:**
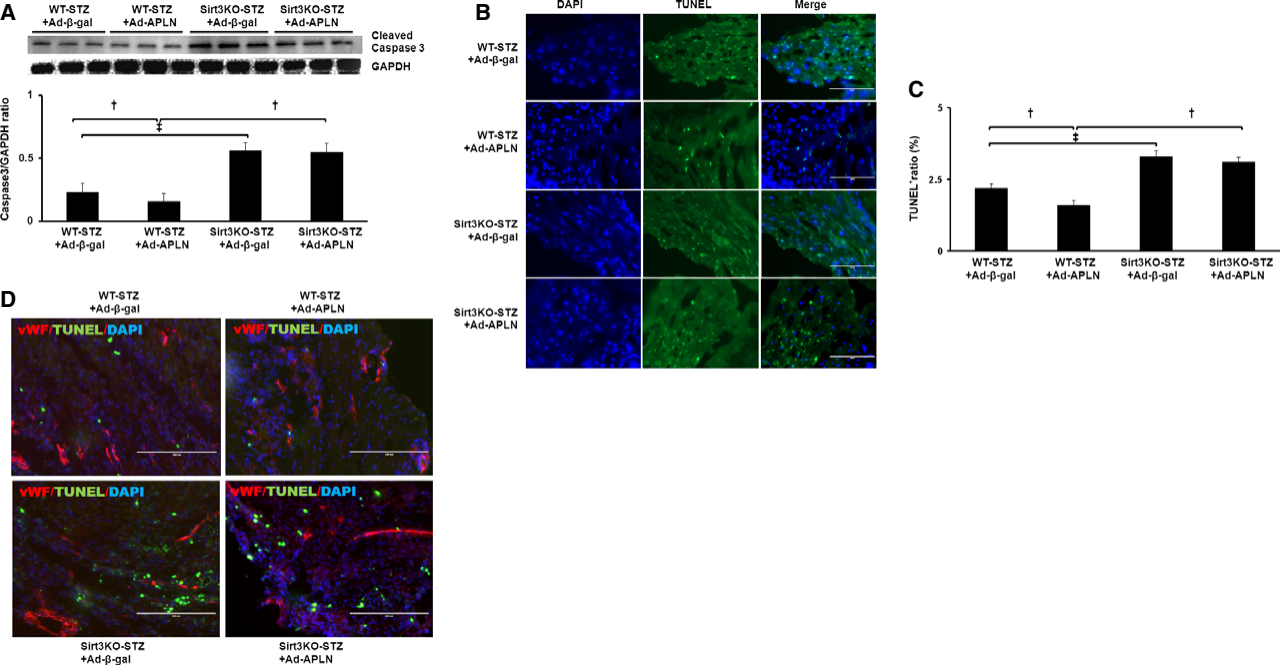
(**A**) The caspase-3 expression was significantly suppressed by Ad-APLN gene therapy in WT-STZ mice compared to Ad-β-gal treatment (*n* = 3, †*P* < 0.001). Sirt3KO-STZ mice had a significant higher caspase-3 expression than WT-STZ at baseline level. APLN gene therapy did not suppress caspase-3 expression in Sirt3KO-STZ mice compared to Ad-β-gal treatment. (**B** and **C**) TUNEL staining showed that the number of TUNEL^+^ cells in the ischaemic border zone was significantly higher in Sirt3KO-STZ mice compared to WT-STZ mice. Ad-APLN gene therapy significantly reduced the number of TUNEL^+^ cells in WT-STZ mice (*n* = 5, †*P* < 0.001). In Sirt3 KO-STZ mice, there were no significant changes in the number of TUNEL^+^ cells in mice received either Ad-APLN or Ad-β-gal treatment. Sirt3 KO-STZ mice had higher number of TUNEL^+^ cells in the heart compared to those of WT-STZ mice at baseline levels (‡*P* < 0.05) and after Ad-APLN treatment (†*P* < 0.001). (**D**) Co-staining of vWF and TUNEL in the heart. vWF was not co-localized with TUNEL in the border zone of ischaemic heart.

Sirt3KO-STZ+MI mice had a significant higher cleaved caspase-3 expression than WT-STZ+MI mice. Myocardial cleaved caspase-3 expression was significantly suppressed by Ad-APLN treatment when compared with Ad-β-gal treatment in WT-STZ mice (Fig. 4A). Knockout of Sirt3 in STZ mice significantly attenuated APLN gene therapy-mediated suppression of cleaved caspase-3 expression. As shown in Figure 4B and C, the number of TUNEL^+^ cells in the ischaemic border zone was significantly higher in Sirt3KO-STZ+MI mice than WT-STZ+MI mice. In comparison with WT-STZ + Ad-β-gal mice, WT-STZ mice received Ad-APLN treatment showed a significant reduction in TUNEL^+^ cells. In contrast, TUNEL^+^ cells were similar between Sirt3KO+Ad-APLN and Sirt3KO+Ad-β-gal mice (Fig. 4B and C). Surprisingly, TUNEL^+^ cells were not co-localized with endothelial marker vWF (Fig. 4D); suggested cardiomyocytes, but not EC, were apoptosis in the border zone of ischaemic heart.

### APLN-mediated improvement of cardiac function is dependent on Sirt3

**Fig. 5:**
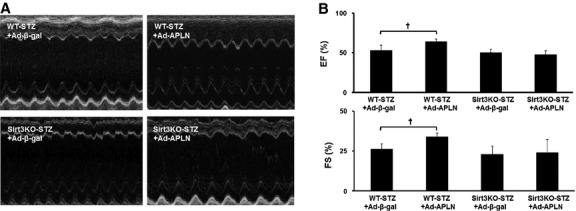
APLN gene therapy improves cardiac function in post-MI STZ mice. Representative images of M mode of echocardiography. Ad-APLN treatment significantly enhanced the post-MI systolic cardiac performance indicated by EF% and FS% in WT-STZ mice compared to Ad-β-gal control injection (*n* = 7, †*P* < 0.001). This improvement of heart function was abolished in Sirt3KO mice (*P* > 0.05).

The echocardiography parameters are shown in Table 1. There was no significant difference in cardiac functional recovery between WT-STZ and Sirt3KO-STZ after MI. The EF% and FS% were significantly improved in WT-STZ + Ad-APLN mice as compared to WT-STZ + Ad-β-gal mice. However, there were no significant differences in EF% and FS% between Sirt3KO-STZ + Ad-APLN mice and Sirt3KO-STZ+Ad-β-gal mice (Fig. 5A and B).

**Table 1:** Cardiac parameters by echocardiography

Variables	WT-STZ+IS +Ad-β-gal	WT-STZ+IS +Ad-APLN	SIRT3KO-STZ+IS +Ad-β-gal	SIRT3KO-STZ+IS +Ad-APLN
ds (mm)	2.69 ± 0.22	2.45 ± 0.21*	2.56 ± 0.25	2.57 ± 0.25
dd (mm)	3.67 ± 0.27	3.73 ± 0.25	3.49 ± 0.1	3.40 ± 0.29
vs (ml/min.)	27.07 ± 5.3	21.53 ± 4.4*,†,‡	26.38 ± 4.1	24.23 ± 5.7
vd (ml/min.)	57.47 ± 10.5	59.69 ± 9.6	50.49 ± 3.6	47.75 ± 4.5
SV (ml)	30.36 ± 6.5	38.13 ± 5.6	24.12 ± 2.1	23.52 ± 2.2
EF (%)	52.81 ± 5.5	64.12 ± 3.2*,†,‡	47.89 ± 5.1	49.71 ± 8.2
FS (%)	26.68 ± 3.5	34.27 ± 2.2*,†,‡	23.51 ± 3.05	24.71 ± 5.1
aws (mm)	0.92 ± 0.12	0.93 ± 0.05	0.89 ± 0.04	0.87 ± 0.15
awd (mm)	0.59 ± 0.09	0.63 ± 0.09	0.64 ± 0.04	0.63 ± 0.12
pws (mm)	0.64 ± 0.08	0.90 ± 0.04*,†,‡	0.73 ± 0.04	0.75 ± 0.08
pwd (mm)	0.44 ± 0.09	0.53 ± 0.05*	0.47 ± 0.07	0.51 ± 0.07

* vs.WT-STZ+IS+Ad-β-gal, *P* < 0.05.

† SIRT3KO-STZ+IS+Ad- β-gal, *P* < 0.05.

‡ SIRT3KO-STZ+IS+Ad-APLN, *P* < 0.05.

ds, diameter systolic; dd, diameter diastolic; vs, volume systolic; vd, volume diastolic; SV, stroke volume; EF, Ejection Fraction; FS, Fractional Shortening; aws, systolic anterior wall thickness; awd, diastolic anterior wall thickness; pws, systolic posterior wall thickness; pwd, systolic posterior wall thickness.

### Overexpression of APLN increases expression of angiogenic growth factors and angiogenesis in EPC, but not EPC from Sirt3KO mice

**Fig. 6:**
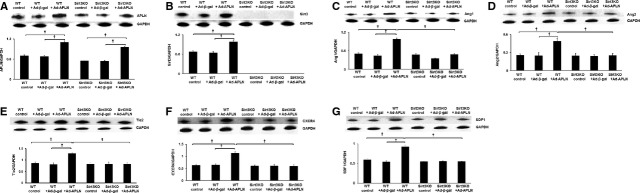
Overexpression of APLN increases expression of angiogenic growth factors and angiogenic capacity of EPC. (**A**) Ad-APLN transfection induced dramatic up-regulation of APLN expression in both WT-EPC and Sirt3KO-EPC (*n* = 3, †*P* < 0.001). (**B**) Overexpression of APLN significantly increased Sirt3 expression in WT-EPC (*n* = 3, †*P* < 0.001). No Sirt3 expression was detected in Sirt3KO-EPC. (**C** and **E**) Compared to Ad-β-gal treatment, Ad-APLN treatment significantly increased in expression of Ang-1 (**C**), Ang-2 (**D**) and Tie-2 (**E**) in WT-EPC. These increases of angiogenic factor expression were demolished in Sirt3KO-EPC (*n* = 3, †*P* < 0.001). (**F** and **G**) Ad-APLN treatment led to significant increase in CXCR4 and SDF1α expression in WT-EPC compared to Ad-β-gal transfection (*n* = 3, †*P* < 0.001). Knockout of Sirt3 in EPC completely inhibited Ad-APLN-induced CXCR-4 and SDF1α expression.

To mimic STZ hyperglycaemic condition *in vivo*, cultured EPCs were exposed to high glucose (30 mmol/l) for 24 hrs before transfection with Ad-APLN. APLN expression was dramatically up-regulated in both WT-EPC and Sirt3KO-EPC by Ad-APLN transfection (Fig. 6A). Overexpression of APLN resulted in a significant increase in Sirt3 expression in WT-EPC. The expression of Sirt3 was not detected in Sirt3KO-EPC (Fig. 6B). Ad-APLN treatment further significantly increased expression of Ang-1, Ang-2 and Tie-2 compared to Ad-β-gal treatment in WT-EPC (Fig. 6C–E). Ad-APLN treatment also led to a significant increase in expression of CXCR4 and SDF1α in WT-EPC. Knockout of Sirt3 in EPC completely abolished APLN-induced expression of angiopoietins/Tie-2 expression as well as CXCR-4 and SDF1α expression (Fig. 6F and G).

**Fig. 7:**
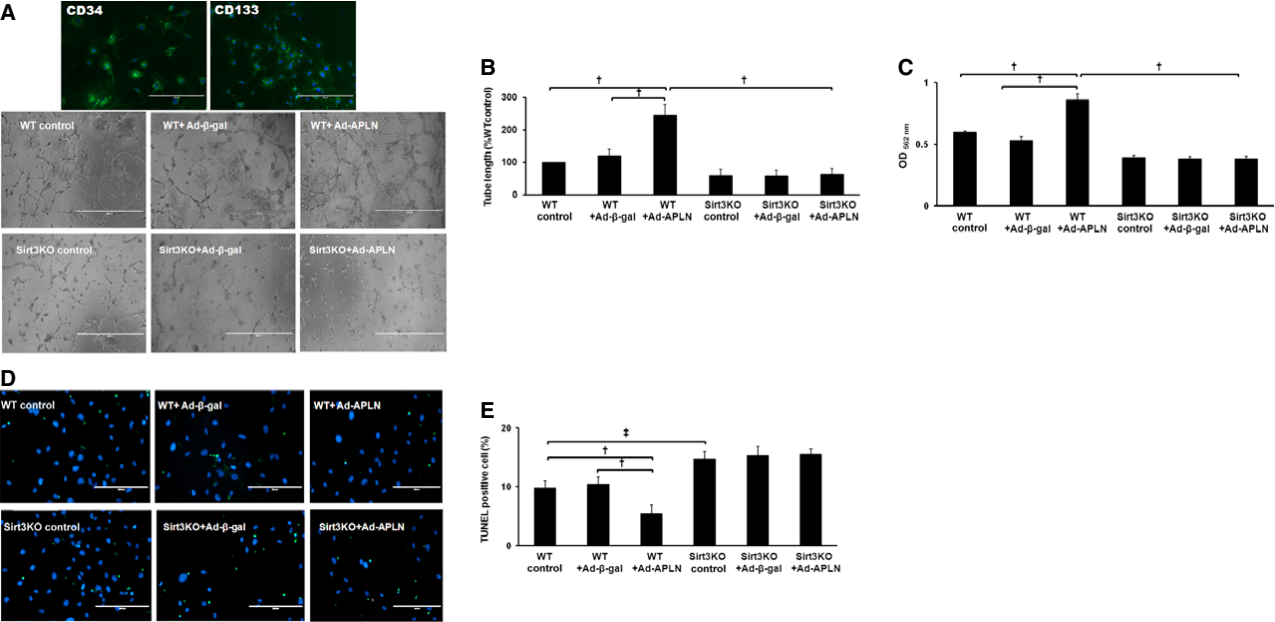
The effect of Ad-APLN on tube formation and proliferation of EPC *in vitro*. (**A** and **B**) Bone marrow EPC was stained with EPC marker CD34 and CD133. Overexpression of APLN in EPC resulted in a significant increase in the number of tube-like structures *in vitro* (*n* = 3, †*P* < 0.001). The increased ability of tube formation was reduced in Sirt3KO-EPC treated with Ad-APLN. (**C**) Overexpression of Ad-APLN increased EPC proliferation as compared to control EPC or EPC treated with Ad-β-gal. Sirt3KO-EPC exhibited a lower proliferation and Ad-APLN treatment failed to increase proliferation of EPC. (**D** and **E**) Overexpression of Ad-APLN reduced the number of TUNEL^+^ cells in EPC under high glucose conditions compared to control cells or cells treated with Ad-β-gal (*n* = 5, †*P* < 0.001). Deficiency of Sirt3 completely abolished the protective effect of APLN against high glucose-induced apoptosis *in vitro*.

Overexpression of APLN further significantly enhanced tube formation and cell proliferation in WT-EPC, but did not in Sirt3KO-EPC (Fig. 7A–C). Moreover, knockout of Sirt3 abolished APLN-mediated suppression of apoptosis in EPC (Fig. 7D and E). Knockout of Sirt3 in EPC significantly blunted APLN-induced suppression of apoptosis under high glucose conditions (Fig. 7D and E).

## Discussion

Our data demonstrated that overexpression of APLN resulted in a significant up-regulation of Sirt3 and angiogenic growth factor expression in post-MI STZ mice. This was accompanied by a significant improvement of myocardial angiogenesis and cardiac function. Intriguingly, knockout of Sirt3 abolished APLN-mediated angiogenesis and cardiac protection in post-MI STZ mice. Our data suggest that APLN gene therapy improves cardiac function by promoting myocardial angiogenesis *via* a mechanism involving up-regulation of Sirt3.

Our present study provides direct evidence that Sirt3 has a critical role in apelin-induced myocardial angiogenesis in ischaemic heart of diabetes. Sirt3 is expressed abundantly in the heart and has been reported to play a protective role in heart. Sirt3 protects cells from stress-mediated death by deacetylation of Ku70 [25] and blocks the cardiac hypertrophic response by augmenting Foxo3a-dependent antioxidant action in mice [26]. Although several recent studies indicate the involvement of Sirt1 in blood vessel formation [27,28], the functional role of Sirt3 in the regulation of angiogenesis especially in ischaemic diabetes has not been reported. APLN has been shown to induce endothelial cells sprouting in an autocrine or paracrine manner, thus contributing to vascular morphogenesis [14,29]. Angiogenesis is mainly regulated by the interplay between VEGF/VEGFR2 and angiopoietins/Tie-2 system. The APLN/APJ and Angs/Tie2 system interaction has been shown to be involved in the vessel calibre size and blood vessel maturation [30]. Ang-1/Tie2 interaction can activate endothelial cells (ECs) to produce endogenous APLN, which contributes to the lumen enlargement of tube-like structures by promoting proliferation and aggregation/assembly of ECs [30]. Our present study revealed that APLN gene therapy increased Angs/Tie-2 and VEGF/VEGFR2 expression together with a significant increase in capillary and arteriole densities in post-MI STZ mice. Interestingly, knockout of Sirt3 completely abolished APLN gene therapy-mediated enhancement of angiogenic factor expression, which resulted in poorer neovascularization *in vivo*. Knockout of Sirt3 further blunted APLN-mediated cardiac function recovery in STZ+MI mice. Based upon these data, we proposed that Sirt3 regulates APLN-mediated angiogenesis in ischaemic heart, at least in part, *via* up-regulation of Angs/Tie-2 and VEGF/VEGFR2 system.

The mobilization of bone marrow-derived EPC has been shown to promote new vessel formation and ameliorates ischaemic injury [31,32]. Both animal and clinical studies showed that augment of EPC mobilization improved angiogenesis in ischaemic area of post-MI [33,34]. CXCR4 and SDF-1α are two key regulators for the mobilization of EPC from bone marrow to the ischaemic heart [35,36]. Our previous study has indicated that APLN promotes cardiac repair by increasing BM-derived vascular progenitor cell homing and stimulating angiogenesis in post-MI mouse heart [18]. Our recent study further demonstrated that APLN-overexpressed bone marrow EPC improves cardiac angiogenesis and functional recovery in post-MI mice *via* activation of Sirt3 signalling pathway [19]. Consistent with our previous studies, our *in vitro* data showed that overexpression of APLN up-regulated CXCR4 and SDF-1α and enhanced angiogenic growth factor expression in EPC. APLN also significantly increased EPC proliferation and tube formation ability. Knockout of Sirt3 in EPC abolished APLN-induced angiogenic growth factor expression and angiogenesis *in vitro*. Moreover, APLN gene therapy increased CXCR4 and SDF-1α expression in WT-STZ post-MI, but not in Sirt3KO-STZ mice. These data suggest that Sirt3 may also be involved in APLN-mediated mobilization of EPC to ischaemic heart *via* SDF-1α/CXCR4 axis in post-MI STZ mice.

Apelin has direct role in cardiomyocyte survival and cardiac contractility. Consistent with these, our co-localization of vWF and TUNEL data suggested a potential involvement of apelin gene therapy on cardiomyocyte apoptosis, but not EC in ischaemic heart of diabetes. Some limitations should be pointed out in this study. Sirt3 is known to improve cardiac metabolisms, limit the cardiac fibrosis and cardiac hypertrophy. In addition, inflammation has an important role in improving the cardiac function more than angiogenesis in post-MI. All these effects, no doubt, contribute to the post-MI function recovery of diabetic hearts. We also recognized that a recent study indicates double-edged role of the CXCL12/CXCR4 axis in experimental MI [37]. In this study, we did not evaluate these effects.

In summary, our study provides direct evidence that overexpression of APLN enhances angiogenesis and improves post-MI cardiac function of STZ mice *via* up-regulation of Sirt3. Our data suggest that modification of Sirt3 with APLN could be used as a novel therapy strategy for the treatment of diabetes-associated impairment of angiogenesis after MI.
